# Bioactivity-Guided Fractionation Identifies Amygdalin as a Potent Neurotrophic Agent from Herbal Medicine *Semen Persicae* Extract

**DOI:** 10.1155/2014/306857

**Published:** 2014-06-24

**Authors:** Chuanbin Yang, Jia Zhao, Yuanyuan Cheng, Xuechen Li, Jianhui Rong

**Affiliations:** ^1^School of Chinese Medicine, Li Ka Shing Faculty of Medicine, The University of Hong Kong, 10 Sassoon Road, Pokfulam, Hong Kong; ^2^Department of Chemistry, The University of Hong Kong, Pokfulam, Hong Kong

## Abstract

Herbal medicine *Semen Persicae* is widely used to treat blood stasis in Chinese medicine and other oriental folk medicines. Although little is known about the effects of *Semen Persicae* and its active compounds on neuron differentiation, our pilot study showed that *Semen Persicae* extract promoted neurite outgrowth in rat dopaminergic PC12 cells. In the present study, we developed a bioactivity-guided fractionation procedure for the characterization of the neurotrophic activity of *Semen Persicae* extract. The resultant fractions were assayed for neurite outgrowth in PC12 cells based on microscopic assessment. Through liquid-liquid extraction and reverse phase HPLC separation, a botanical glycoside amygdalin was isolated as the active compound responsible for the neurotrophic activity of *Semen Persicae* extract. Moreover, we found that amygdalin rapidly induced the activation of extracellular-signal-regulated kinase 1/2 (ERK1/2). A specific ERK1/2 inhibitor PD98059 attenuated the stimulatory effect of amygdalin on neurite outgrowth. Taken together, amygdalin was identified as a potent neurotrophic agent from *Semen Persicae* extract through a bioactivity-guided fractional procedure. The neurotrophic activity of amygdalin may be mediated by the activation of ERK1/2 pathway.

## 1. Introduction

Neurotrophic factors including nerve growth factor (NGF) and brain-derived neurotrophic factor (BDNF) regulate the survival, growth, and repair of neurons, thereby maintaining the integrity of neurons [1–3]. Recently, neurotrophic factors appear to hold great promise in treating not only acute neuronal injuries due to trauma and stroke, but also chronic neurodegenerative diseases including Alzheimer's disease (AD), Parkinson's disease (PD), and amyotrophic lateral sclerosis (ALS) [1, 4, 5]. However, the therapeutic applications of neurotrophic factors are largely hindered by the lack of efficient delivery into the brains and the presence of undesirable side effects [5, 6]. Thus, small molecules capable of mimicking the actions of these neurotrophic factors may serve as an alternative therapeutic strategy for neurological diseases [7]. Rat pheochromocytoma cell line PC12 is widely used as the cell model for* in vitro* studies of neuronal differentiation and the underlying mechanisms [8]. For example, NGF induced the differentiation of PC12 cells into functional dopaminergic neurons [9]. Mechanistic studies revealed that NGF induced neurite outgrowth and neuronal differentiation through sustained activation of extracellular signal-regulated kinase 1/2 (ERK1/2) by interacting with its specific receptor tyrosine kinase (TrkA) in PC12 cells [10, 11].

Herbal medicines are used as an enormous source for drug discovery either as lead compounds or candidate drugs [12, 13]. Herbal medicine* Semen Persicae*, the fruit-kernel of peach, is commonly used to treat blood stagnant syndrome such as anticoagulant, antiphlogistic, and anodyne in traditional Chinese medicine [14, 15]. Interestingly,* Semen Persicae* is also used in many herbal medicine formulations including Bu-Yang-Huan-Wu-Tang (also designated as ISF-1) for the treatment of poststroke disorders [16, 17]. However, little is known about the pharmacological effects of* Semen Persicae* on neuronal differentiation.

In the present study, our initial study showed that* Semen Persicae* extract promoted neurite outgrowth in PC12 cells. Thus, we developed a bioactivity-guided fractionation procedure for rapid identification of the neurotrophic agent from* Semen Persicae* extract. We also attempted to elucidate the potential mechanisms underlying the neurotrophic activities of the active compound derived from* Semen Persicae* extract.

## 2. Materials and Methods

### 2.1. Chemical and Reagents

The dried powder of herbal medicine* Semen Persicae* extract was purchased from a local Pharmaceutical company Nong's Company (Hong Kong). Antibodies against ERK1/2, phospho-ERK1/2, *β*3-tubulin, goat anti-rabbit IgG secondary antibody, and Alexa Fluor 594-conjugated goat anti-rabbit IgG antibody were obtained from Cell Signaling Technology (Boston, MA, USA). Monoclonal antibodies against growth associated protein-43 (GAP-43), microtubule associated protein 2 (MAP2), and glyceraldehyde-3-phosphate dehydrogenase (GAPDH) were purchased from Santa Cruz Biotechnology (Santa Cruz, CA, USA). Fluorescein isothiocyanate conjugated phalloidin (FITC-phalloidin) and other chemicals were obtained from Sigma-Aldrich Co. (St. Louis, MO, USA) unless indicated otherwise.

### 2.2. Cell Culture and Neurite Outgrowth Assay

Rat pheochromocytoma cell line PC12 was obtained from the American Type Cell Culture Collection (Manassas, VA, USA) and maintained in growth medium (GM) containing Dulbecco's modified Eagle's medium (DMEM) supplemented with 10% horse serum (HS), 5% fetal bovine serum (FBS), and 1% penicillin/streptomycin on collagen I-coated dishes at 37°C in a humidified 5% CO_2_ atmosphere. Neurite outgrowth was assayed essentially as described [18]. Briefly, the cells were seeded in growth medium. After overnight incubation, the cell culture medium was replaced with differentiation medium containing 1% HS and 0.5% FBS in DMEM. The fractions and testing compounds were assayed for neurite outgrowth. Following 3 days of incubation, the neurite-bearing cells were examined and the cells bearing neurites longer than 20 *μ*m were enumerated under a phase microscope from Carl Zeiss (Göttingen, Germany).

### 2.3. High Performance Liquid Chromatography (HPLC) Analysis

HPLC analysis was performed on a COSMOSIL HILIC C18 column (250 mm × 4.6 mm i.d.) under the control of a Waters HPLC system. This system was equipped with a Waters 996 Model photodiode array detector (DAD), a Waters 600S Model system controller, and a gradient generator. The column was operated in a gradient mobile phase by mixing an aqueous solution (A) and acetonitrile (B). The samples were eluted by 5% B for the first 5 min, and followed by a linear gradient from 5% to 95% B over 50 minutes at the flow rate of 1 mL/min, and the column was maintained at room temperature. The elution was monitored by continuously recording the UV absorbance at 210 nm.

### 2.4. Bioactivity-Guided Fractionation of* Semen Persicae* Extract

Liquid-liquid extraction: twenty grams of the dried* Semen Persicae* extract were resuspended in 100 mL of Millipore water and heated at 80°C for 1 hour. Following the removal of the insoluble materials by centrifugation, the supernatant (A) was recovered and sequentially extracted three times with 100 mL of ethyl acetate and n-butanol. The solvent of each extraction was removed on a Büchi rotary evaporator under vacuum. The dried residues from each preparation were dissolved in DMSO and sterilized by passing through a 0.22 *μ*m membrane filter for the biological assays.

Semipreparative HPLC separation: For the isolation of the active compound, the n-butanol fraction was further separated by semipreparative reverse phase HPLC on an Alltech Alltima C-18 column (10.0 mm I.D. × 250 mm, 5 *μ*m). The column was run in a gradient mobile phase generated by mixing an aqueous solution (A) and acetonitrile (B). The samples were eluted by 5% of B for 5 minutes and followed by a linear gradient elution with up to 95% of B over the period of 50 minutes. The flow rate was set at 3 mL/min and column temperature was maintained at 25°C. The UV absorbance was monitored at the 200 nm to 400 nm wavelengths. A total of nine fractions were recovered and assayed for neurite outgrowth in PC12 cells.

### 2.5. Chemical Identification by Mass Spectrometry (MS)

The HPLC fraction containing the active compound was analyzed by HPLC-MS on a Varian Microsorb C18 column (2 × 150 mm) at the flow rate of 0.8 mL/min under the control of a Waters HPLC system equipped with a 1525 Separations Module and a Waters 2998 DAD Unit. Mobile phase compositions were (A) water with 0.3% (v/v) formic acid and (B) acetonitrile. Gradient was set as follows: 0–8 min, 5–50% B; 8–10 min, 50% B; 10–12 min, 50–90% B; 12–15 min, 95% B. Flow rate was constant at 0.8 mL/min. The column temperature was maintained at 20°C. Injection volume was 20 *μ*L. The eluents were analyzed on an ABI/Sciex triple quadrupole mass spectrometer 3200 QTRAP system (Framingham, MA, USA) equipped with an ESI-Turbo V source operating in positive ionization mode under the control of Analyst v1.4.2 data system (Applied Biosystems/MDS Sciex, Concord, ON, Canada). Mass spectrometry was operated under the conditions as follows: drying gas N_2_, 10 L/min; capillary voltage, 20 v; pressure of nebulizer, 30 psi; ion spray voltage, 4 kV; and capillary temperature, 325°C.

### 2.6. Immunocytochemical Staining

At the end of drug treatment, the cells were fixed in 3.7% formaldehyde (PFA), permeabilized in 0.5% Triton X-100, and blocked in 5% normal goat serum. The cells were then incubated with FITC-phalloidin and anti-MAP2 antibody overnight at 4°C, and the bound antibodies were detected with an Alexa Fluor 594-conjugated goat anti-rabbit IgG secondary antibody. The cell nuclei were visualized by staining with 4′-6-diamidino-2-phenylindole (DAPI). The fluorescence images were acquired on a fluorescence microscope from Carl Zeiss (Göttingen, Germany).

### 2.7. Western Blotting Analysis

At the end of drug treatment, the cellular proteins were recovered and analyzed by Western blotting as previously described [19]. Briefly, the cellular proteins were resolved by electrophoresis on 10% SDS-PAGE gels and subsequently transferred onto polyvinylidenedifluoride (PVDF) membrane. After being blocked with 5% nonfat milk powder in TBS buffer, the blots were probed with the antibodies specific for the indicated proteins. The primary antibodies on the blots were detected with a goat anti-rabbit IgG-HRP conjugate. The activity of peroxidase was assayed with enhanced chemiluminescence (ECL) detection reagents from GE Healthcare (Uppsala, Sweden) according to the manufacturer's instruction.

### 2.8. Statistical Analysis

The results were presented as means ± SD and analyzed by the paired one-tailed *t*-test. A *P* value of <0.05 was considered statistically significant.

## 3. Results

### 3.1. *Semen Persicae* Extract Induces Neurite Outgrowth

**Figure 1:**
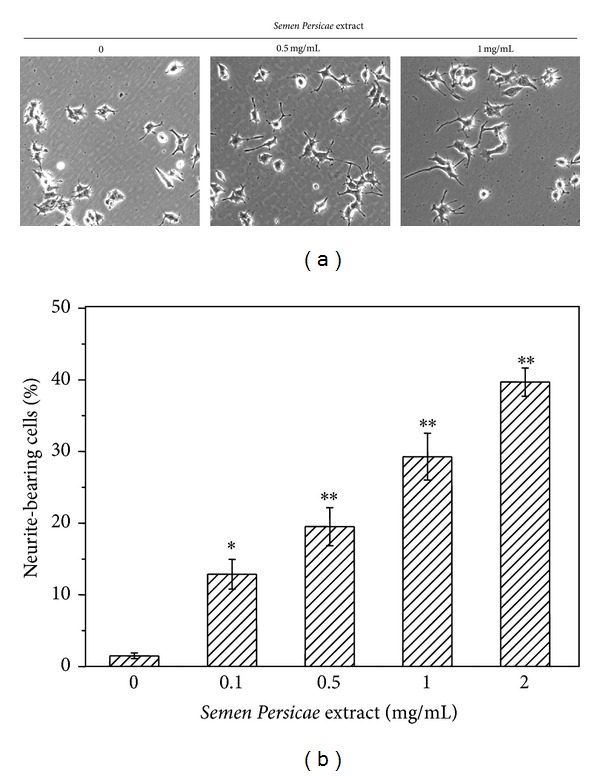
The neurotrophic effect of* Semen Persicae* extract. (a) Induction of neurite outgrowth by* Semen Persicae* extract. PC12 cells were treated with* Semen Persicae* extract at the concentrations of 0, 0.1, 0.5, 1, and 2 mg/mL for 3 days. The cell morphology was examined under a microscope and representative figures are shown. (b) Quantification of the neurite bearing cells in (a). Data are presented as mean ± SE. *P < 0.05 Treatment versus Control.

The neurotrophic effect of* Semen Persicae* extract was evaluated as previously described [18]. Following the exposure to the aqueous extract of* Semen Persicae* at the concentrations of 0, 0.1, 0.5, 1, and 2 mg/mL for 72 hours, the neurite-bearing cells were examined and enumerated under a microscope. As shown in Figures 1(a) and 1(b),* Semen Persicae* extract induced PC12 neurite outgrowth in a concentration-dependent manner.

### 3.2. Bioactivity-Guided Fractionation for the Identification of the Neurotrophic Agent from* Semen Persicae* Extract

**Figure 2:**
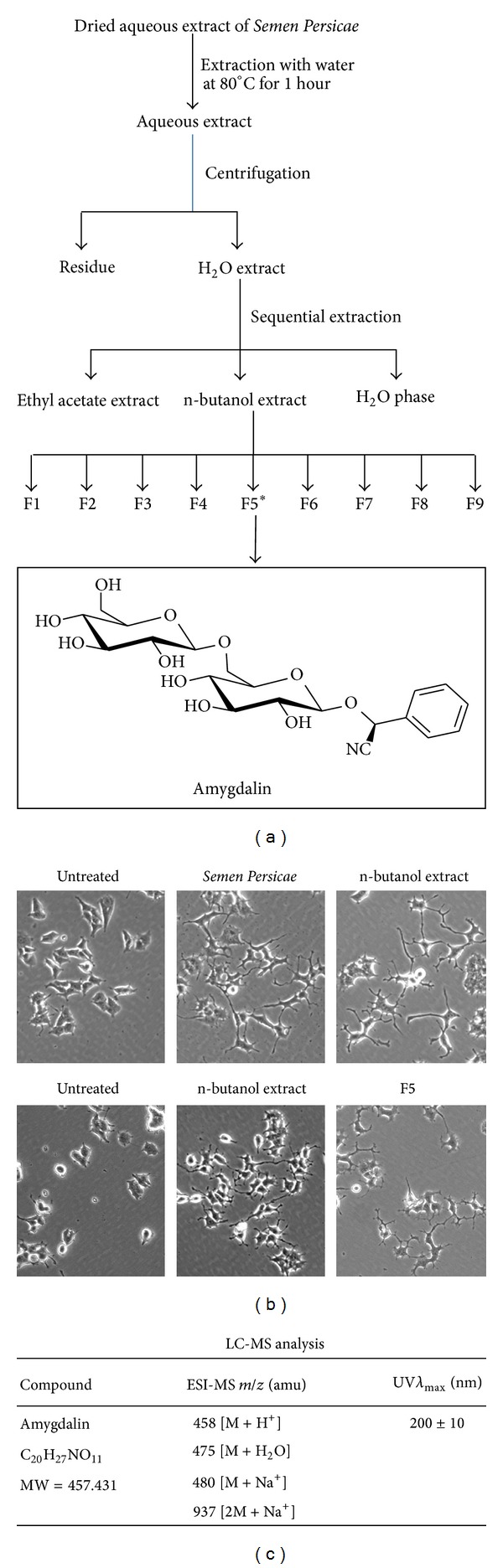
Bioactivity-guided fractionation for the identification of the neurotrophic agent from* Semen Persicae* extract. (a) Scheme illustrating the bioactivity-guided fractionation procedure for the isolation of botanical glycoside amygdalin as the neurotropic compound from* Semen Persicae* extract. The structure of amygdalin was generated by ChemSketch software (http://www.acdlabs.com). (b, upper panel) Assay of the fractions generated by liquid-liquid extraction. The aqueous* Semen Persicae* extract was sequentially extracted by ethyl acetate, n-butanol, giving rise to three fractions: ethyl acetate extract, n-butanol extract, and H_2_O phase. These fractions were assayed for the induction of neurite outgrowth in PC12 cells. The images were acquired under a microscope. (b, lower panel) Assay of the fractions generated by semipreparative HPLC separation and representative images are shown. The n-butanol extract was separated into a total of 9 fractions by semipreparative HPLC isolation. All HPLC fractions were assayed for the induction of neurite outgrowth in PC12 cells. The images were acquired under a microscope and F5 with neurotrophic activities are shown. (c) ESI-MS analysis and chemical information of the active compound. ESI-MS ions (*m*/*z*) were identified by HPLC-MS analysis as described in Section 2. The ultraviolet (UV) absorption spectrum was recorded by HPLC UV detector for the identification of UV *λ*_max⁡_ (nm).

To characterize the active compound responsible for promoting neurite outgrowth, we developed a bioactivity-guided fractionation procedure for the identification of the neurotrophic agent from* Semen Persicae* extract (Figure 2(a)). The aqueous extract of* Semen Persicae* was sequentially extracted with ethyl acetate and n-butanol, giving rise to three new fractions, namely, ethyl acetate extract, n-butanol extract, and H_2_O phase. All these fractions were evaluated for the promotion of neurite outgrowth. As shown in Figure 2(b) (upper panel), n-butanol extract effectively promoted neurite outgrowth.

**Figure 3:**
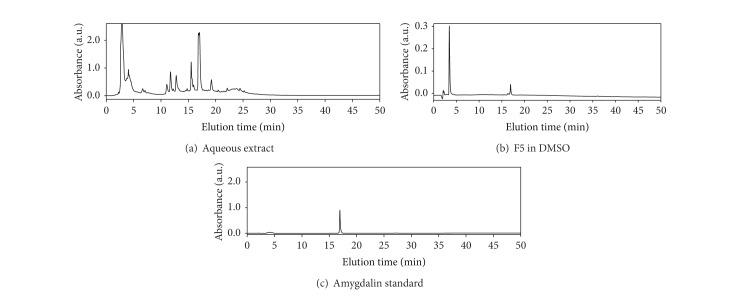
Representative HPLC chromatographic profiles. (a) HPLC chromatographic profile of n-butanol extract. (b) HPLC chromatographic profile of F5. (c) HPLC chromatographic profile of commercial amygdalin. HPLC chromatographic analyses were performed in a similar fashion. The HPLC fractions were monitored by recording the UV absorbance at 210 nm.

To characterize the active compound from the n-butanol extraction, the n-butanol extraction was separated by HPLC on a C18 column into 9 different fractions based on the pilot experiment (Figure 2(a)). All these fractions were examined for the activity of promoting neurite outgrowth in PC12 cells. As shown in Figure 2(b) (bottom panel), Fraction Number 5 (F5) showed the strongest activity in promoting neurite outgrowth. The HPLC profile of the n-butanol extraction was shown in Figure 3(a), whereas F5 exhibited almost a single peak profile in another HPLC analysis under the same conditions (Figure 3(b)). Therefore, F5 was directly characterized by HPLC-MS technology. The positive ESI-MS *m*/*z* spectrum of F5 showed four major signals including 458 [M + H^+^], 457 [M], 480 [M + Na^+^], and 937 [2M + Na^+^]. The HPLC-MS analysis suggested that F5 could be botanical glycoside amygdalin (Figure 2(c)). In addition, the ultraviolet (UV) absorption spectrum (data not shown) suggested that UV *λ*_max⁡_ (nm) was corresponding to 200 ± 10 nm. To verify the chemical identity of F5, we obtained the HPLC profile for commercial amygdalin (Figure 3(c)). Remarkably, F5 was eluted in the identical profile as the commercial amygdalin (Figures 3(b) and 3(c)).

### 3.3. Amygdalin Induced Neurite Outgrowth in a Concentration-Dependent Manner

**Figure 4:**
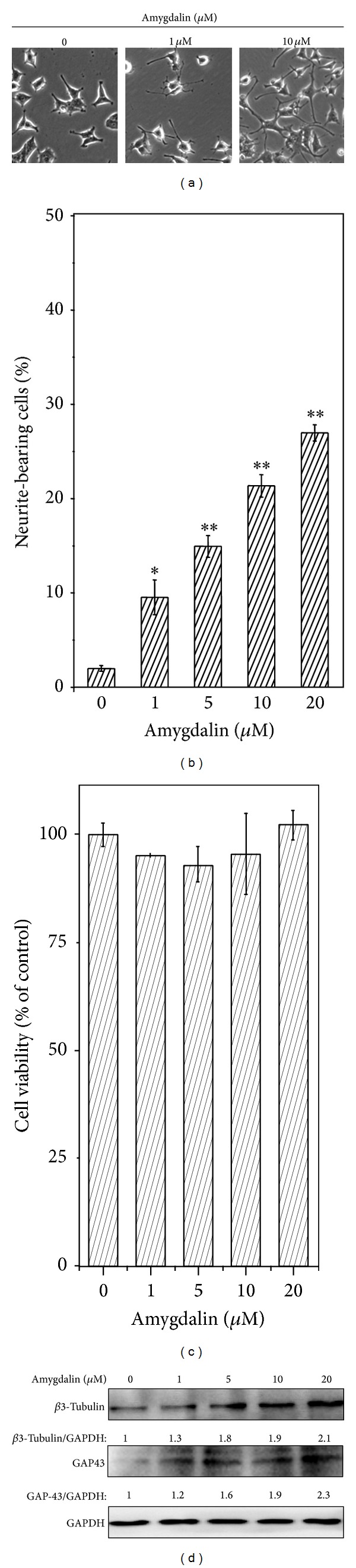
Evidence for the neurotrophic activity of amygdalin. (a) The neurotrophic activity of amygdalin. PC12 cells were treated with amygdalin at concentration ranging from 0 to 20 *μ*M for 72 h. The neurite outgrowth was examined under a microscope. The representative images were shown. (b) Quantitative analysis of amygdalin-induced neurite outgrowth. Data are presented as mean ± SD (*n* = 3). *P < 0.05 (Treatment versus Control). (c) Effect of amygdalin on the cell viability. PC12 cells were treated as described in Panels (a) and (b). The cell viability was determined by standard MTT assay (*n* = 3). (d) Effect of amygdalin on the expression of neuronal biomarkers. At the end of drug treatment, neuronal biomarkers were detected by Western blotting as described in Section 2. Three independent experiments were performed. The mean values for fold of induction were shown under each treatment.

To confirm the possibility of amygdalin as the active compound, we first treated PC12 cells with commercial amygdalin at the concentrations of 0, 1, 5, 10, and 20 *μ*M for 72 hours. The neurite outgrowth was examined under a microscope. As shown in Figures 4(a) and 4(b), amygdalin induced neurite outgrowth in a concentration-dependent manner. Secondly, we examined if amygdalin could affect the cell viability of PC12 cells.  Figure 4(c) clearly showed that amygdalin would be toxic to the cells at the concentration up to 20 *μ*M. Thirdly, the neuronal biomarkers *β*3-tubulin and GAP43 were examined by Western blotting using specific antibody, whereas GAPDH was also detected as the control of protein loading. As shown in Figure 4(d), amygdalin increased the expression of *β*3-tubulin and GAP43 in a concentration-dependent manner.

### 3.4. Amygdalin Induced Neurite Outgrowth via Activating ERK1/2 Pathway

**Figure 5:**
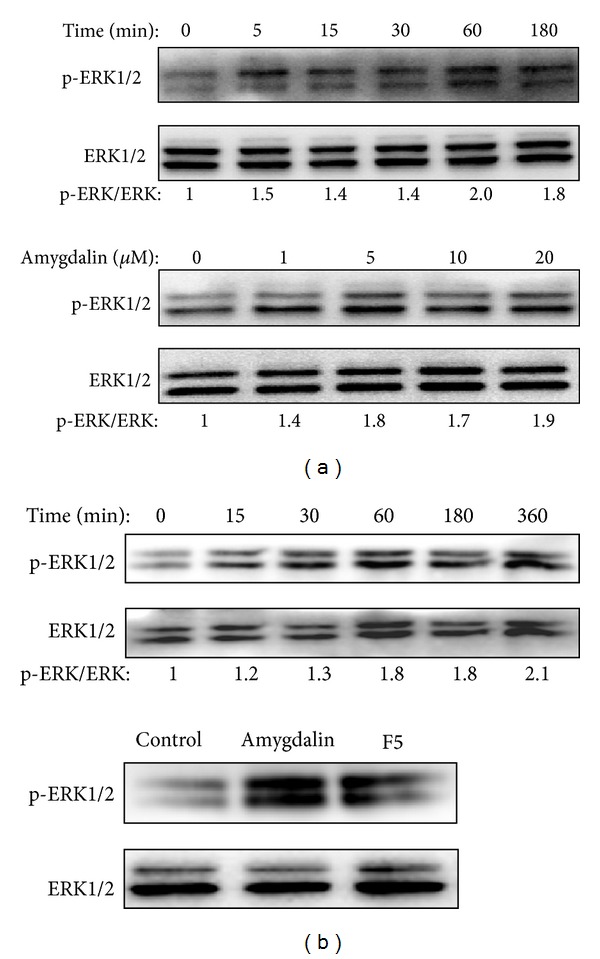
Activation of ERK1/2 pathway by amygdalin and amygdalin-containing fractions. (a) Effect of amygdalin on the phosphorylation of ERK1/2. PC12 were treated with 20 *μ*M of amygdalin for indicated times or different concentrations of amygdalin (0–20 *μ*M) for 1 hour. Total and phosphorylated ERK1/2 were detected by Western blotting analysis. (b) Effect of amygdalin-containing fractions on the phosphorylation of ERK1/2. For upper panel, PC12 were treated with* Semen Persicae* extract (1 mg/mL) for indicated times. For lower panel, PC12 were treated with amygdalin (20 *μ*M) or F5 (1 mg/mL relative to the parent* Semen Persicae* extract) for 60 minutes. Total and phosphorylated ERK1/2 were detected by Western blotting analysis. Three independent experiments were performed. The mean values for fold of induction were shown under each treatment.

**Figure 6:**
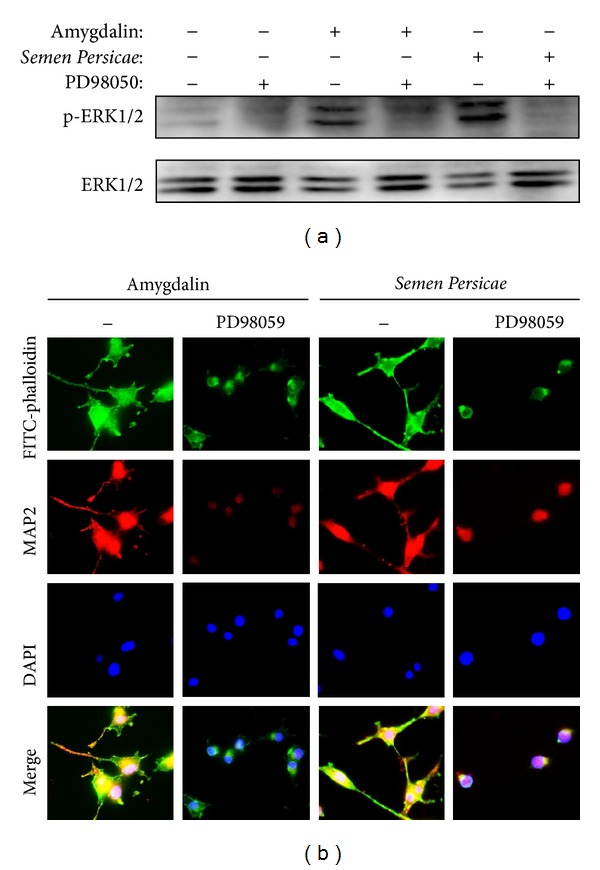
Potential role of ERK1/2 in the neurotrophic activity of amygdalin. (a) Effect of MEK inhibitor PD98059 on the activation of ERK1/2 by amygdalin and* Semen Persicae* extract. PC12 were incubated with amygdalin (20 *μ*M) or* Semen Persicae* extract (1 mg/mL) at the presence or absence of PD98059 (20 *μ*M) for 60 minutes. Total and phosphorylated ERK1/2 were detected by Western blotting analysis. (b) Effect of MEK inhibitor PD98059 on the neurotrophic activity of amygdalin and* Semen Persicae* extract. After 3 days of incubation with amygdalin (20 *μ*M) or* Semen Persicae* extract (1 mg/mL) at the presence or absence of PD98059 (20 *μ*M), the cells were stained with FITC-phalloidin for F-actin, anti-MAP2 for neuronal characteristics, and DAPI for cell nucleus. The images were acquired under a fluorescence microscope.

To further investigate the potential molecular mechanism by which amygdalin induced neurite outgrowth, we first examined the effect of amygdalin on the phosphorylation of ERK1/2 in PC12 cell. As shown in Figure 5(a), amygdalin induced the activation of ERK1/2 in a time- and concentration-dependent manner. Secondly, we verified that* Semen Persicae* extract also induced the activation of ERK1/2 in a similar fashion, whereas F5 and amygdalin showed comparable activity in the activation of ERK1/2 (Figure 5(b)). Thirdly, we determined the effect of MEK inhibitor PD98059 on the activation of ERK1/2 in response to the treatment of amygdalin and* Semen Persicae* extract. As expected, MEK inhibitor PD98059 profoundly inhibited the activity of amygdalin and* Semen Persicae* extract on the phosphorylation of ERK1/2 (Figure 6(a)). Finally, we explored the role of ERK1/2 activation in the neurotrophic activity of amygdalin and* Semen Persicae* extract. We cotreated the cells with amygdalin and* Semen Persicae* extract in the absence or presence of PD98059, a specific ERK1/2 inhibitor for 3 days. The cells were stained with FITC-Phalloidin for F-actin, anti-MAP2 for neuronal characteristics, and DAPI for cell nucleus. As shown in Figure 6(b), ERK1/2 inhibitor PD98059 almost abolished the stimulatory effect of amygdalin and* Semen Persicae* extract on the expression of neuronal biomarker MAP2 and the formation of F-actin assembly.

## 4. Discussion

Herbal medicines are emerging treatments for chronic neurodegenerative diseases [20, 21]. Molecular characterization of the active compounds from complex herbal medicines is the key to assess the therapeutic potential of herbal medicines. We recently attempted to integrate the bioactivity-guided fractionation method with genome-wide biological response fingerprinting (BioReF). Compared with the single target-based bioactivity-guided fractionation, our BioReF-based strategy guarantees the high success rate for the identification of the active compounds from complex herbal medicines. For example, we have successfully identified the active compounds for inducing heme oxygenase-1 (HO-1) and leukotriene B4 12-hydroxydehydrogenase (LTB4DH) from the well-known poststroke rehabilitation formulation ISF-1 [22, 23]. In the present study, we investigated the activity of herbal medicine* Semen Persicae* in promoting neurite outgrowth in PC12 cells. We developed a bioactivity-guided fractionation procedure for the identification of amygdalin as a potent neurotrophic compound from herbal medicine* Semen Persicae*.

Therapeutic promotion of adult neurogenesis is crucial for the treatment of various neurodegenerative diseases [3, 24, 25]. It is well known that NGF regulates the survival and differentiation of rat dopaminergic PC12 cells [9]. As a result, this cell line is often used as an important cell model for the study of neuronal differentiation [26]. The neuronal differentiation of PC12 cells is typically evaluated by enumerating the neurite-bearing cells or measuring the average neurite length [18]. In the present study, we investigated the neurotrophic effect of herbal medicine* Semen Persicae* by robust examination of the cell morphology under a microscope. In our pilot study, we treated PC12 cells with* Semen Persicae* extract at different concentrations for 3 days. We found that* Semen Persicae* extract induced neurite outgrowth in a concentration-dependent manner. This finding stimulated us to develop a bioactivity-guided fractionation procedure for rapid identification of the neurotrophic agent from* Semen Persicae* extract. Our procedure involved liquid-liquid extraction and reverse phase HPLC separation (Figure 2(a)). Firstly, we found that the active compound(s) was mainly extracted into n-butanol fraction (Figure 2(b)). Subsequently, n-butanol fraction was separated into a total of nine fractions by semipreparative RP-HPLC. Based on the assay of neurite outgrowth, F5 showed strongest activity in promoting neurite outgrowth (Figure 2(b)). HPLC analysis confirmed that F5 was eluted as a single peak (Figure 3(b)). Thus, we directly analyzed F5 by HPLC-MS technology on a C18 column. The *m*/*z* signals (e.g., 458 [M + H^+^], 457 [M], 480 [M + Na^+^], and 937 [2M + Na^+^]) suggested that the active compound could be amygdalin. We observed that F5 and amygdalin were eluted from the column in a highly similar manner. Importantly, commercial amygdalin not only induced neurite outgrowth but also increased the expression of multiple neuronal biomarkers (e.g., *β*3-tubulin, GAP43, and MAP-2). Based on the microscopic assay of the neurotrophic activity in Figures 1(b) and 4(b), we could roughly estimate that 1 mg/mL of* Semen Persicae* extract contains 20 *μ*M of amygdalin. Amygdalin at the concentrations of 1, 5, 10, and 20 *μ*M did not show any cytotoxicity in PC12 cells. Collectively, we have successfully isolated botanical glycoside amygdalin as the active compound responsible for the neurotrophic activity of* Semen Persicae* extract.

Amygdalin is a naturally occurring glycoside initially isolated from the seeds of* Prunus dulcis*, also known as bitter almonds as early as 1830 [27]. Amygdalin is also present in several other related species such as apples, apricots, almonds, cherries, plums, and peaches [28–30]. Previous studies have demonstrated that amygdalin exhibited a wide range of biological activities including anti-inflammatory, anticancer, antihypertension, and antiatherosclerosis activities [27, 31–33]. However, the neurotrophic effect of amygdalin has not been fully investigated. To our knowledge, this study for the first time demonstrated that amygdalin could effectively induce neurite outgrowth in an* in vitro* cell model of neuronal differentiation.

ERK1/2 pathway is an important signaling pathway that governs cell differentiation, cell survival, and cell cycle [34, 35]. Previous studies suggested that various natural compounds such as fisetin, quercetin promoted neurite outgrowth via the activation of ERK1/2 pathway [36, 37]. In the present study, we found that amygdalin and* Semen Persicae* extract induced the phosphorylation of ERK1/2 in a concentration- and time-dependent manner. F5 and amygdalin showed similar activity to induce the activation of ERK1/2. Importantly, ERK1/2 specific inhibitor PD98059 almost abolished the effect of amygdalin and* Semen Persicae* extract on neurite outgrowth in PC12 cells as evidenced by staining F-actin with FITC-phalloidin and neuronal biomarker MAP2 with specific antibody. We also demonstrated that ERK1/2 specific inhibitor PD98059 profoundly suppressed the activity of amygdalin and* Semen Persicae* extract on the phosphorylation of ERK1/2. These results suggest that amygdalin also induced neurite outgrowth via activating ERK1/2 pathway. Therefore, the activation of ERK1/2 may be a common mechanism supporting the neurotrophic activities of a variety of small molecules and neurotrophins. It is well known that multiple mechanisms could lead to the activation of ERK1/2. Therefore, future work should be directed to the elucidation of the exact mechanism by which amygdalin activates ERK1/2 pathway and the evaluation of the* in vivo* neurotrophic activities of amygdalin.

## 5. Conclusions

In conclusion, we developed a bioactivity-guided fractionation procedure for rapid identification of amygdalin as a potent neurotrophic agent from herbal medicine* Semen Persicae*. Mechanistic studies revealed that the neurotropic effect of amygdalin might be mediated by the activation of ERK1/2 pathway. Thus, amygdalin may serve as a promising lead candidate for the development of new drugs against neurodegenerative diseases.

## References

[1] Allen SJ, Watson JJ, Dawbarn D (2011). The neurotrophins and their role in Alzheimer’s disease. *Current Neuropharmacology*.

[2] Hennigan A, O’Callaghan RM, Kelly ÁM (2007). Neurotrophins and their receptors: roles in plasticity, neurodegeneration and neuroprotection. *Biochemical Society Transactions*.

[3] Sullivan AM, Toulouse A (2011). Neurotrophic factors for the treatment of Parkinson’s disease. *Cytokine & Growth Factor Reviews*.

[4] Bartus RT, Baumann TL, Brown L, Kruegel BR, Ostrove JM, Herzog CD (2013). Advancing neurotrophic factors as treatments for age-related neurodegenerative diseases: developing and demonstrating “clinical proof-of-concept” for AAV-neurturin (CERE-120) in Parkinson’s disease. *Neurobiology of Aging*.

[5] Allen SJ, Watson JJ, Shoemark DK, Barua NU, Patel NK (2013). GDNF, NGF and BDNF as therapeutic options for neurodegeneration. *Pharmacology & Therapeutics*.

[6] Spedding M, Gressens P (2008). Neurotrophins and cytokines in neuronal plasticity. *Novartis Foundation Symposium*.

[7] Price RD, Milne SA, Sharkey J, Matsuoka N (2007). Advances in small molecules promoting neurotrophic function. *Pharmacology & Therapeutics*.

[8] Greene LA, Aletta JM, Rukenstein A, Green SH (1987). PC12 pheochromocytoma cells: culture, nerve growth factor treatment, and experimental exploitation. *Methods in Enzymology*.

[9] Greene LA, Tischler AS (1976). Establishment of a noradrenergic clonal line of rat adrenal pheochromocytoma cells which respond to nerve growth factor. *Proceedings of the National Academy of Sciences of the United States of America*.

[10] Vaudry D, Stork PJS, Lazarovici P, Eiden LE (2002). Signaling pathways for PC12 cell differentiation: making the right connections. *Science*.

[11] Harrington AW, St. Hillaire C, Zweifel LS (2011). Recruitment of actin modifiers to TrkA endosomes governs retrograde NGF signaling and survival. *Cell*.

[12] Harvey AL (2008). Natural products in drug discovery. *Drug Discovery Today*.

[13] Mishra BB, Tiwari VK (2011). Natural products: an evolving role in future drug discovery. *European Journal of Medicinal Chemistry*.

[14] Xu LM, Liu P, Liu C (1994). Observation on the action of extractum semen Persicae on anti-fibrosis of liver. *China journal of Chinese Materia Medica*.

[15] Zhu JL, Liu C (1992). Modulating effects of extractum semen Persicae and cultivated Cordyceps hyphae on immuno-dysfunction of inpatients with posthepatitic cirrhosis. *Chinese Journal of Integrated Traditional and Western Medicine*.

[16] Rong J, Tilton R, Shen J (2007). Genome-wide biological response fingerprinting (BioReF) of the Chinese botanical formulation ISF-1 enables the selection of multiple marker genes as a potential metric for quality control. *Journal of Ethnopharmacology*.

[17] Liu L, Duan J-A, Tang Y (2012). Taoren-Honghua herb pair and its main components promoting blood circulation through influencing on hemorheology, plasma coagulation and platelet aggregation. *Journal of Ethnopharmacology*.

[18] Smit M, Leng J, Klemke RL (2003). Assay for neurite outgrowth quantification. *BioTechniques*.

[19] Yang CB, Pei WJ, Zhao J, Cheng YY, Zheng XH, Rong JH (2014). Bornyl caffeate induces apoptosis in human breast cancer cells in vitro via the ROS-and JNK-mediated pathways. *Acta Pharmacologica Sinica*.

[20] Mercer LD, Kelly BL, Horne MK, Beart PM (2005). Dietary polyphenols protect dopamine neurons from oxidative insults and apoptosis: investigations in primary rat mesencephalic cultures. *Biochemical Pharmacology*.

[21] Chaturvedi RK, Shukla S, Seth K (2006). Neuroprotective and neurorescue effect of black tea extract in 6-hydroxydopamine-lesioned rat model of Parkinson’s disease. *Neurobiology of Disease*.

[22] Qi H, Siu SO, Chen Y (2010). Senkyunolides reduce hydrogen peroxide-induced oxidative damage in human liver HepG2 cells via induction of heme oxygenase-1. *Chemico-Biological Interactions*.

[23] Wei L, Liu J, Le XC (2011). Pharmacological induction of leukotriene B4-12-hydroxydehydrogenase suppresses the oncogenic transformation of human hepatoma HepG2 cells. *International Journal of Oncology*.

[24] Winner B, Kohl Z, Gage FH (2011). Neurodegenerative disease and adult neurogenesis. *European Journal of Neuroscience*.

[25] Gallarda BW, Lledo P-M (2012). Adult neurogenesis in the olfactory system and neurodegenerative disease. *Current Molecular Medicine*.

[26] Satoh T, Nakamura S, Taga T (1988). Induction of neuronal differentiation in PC12 cells by B-cell stimulatory factor2/interleukin 6. *Molecular and Cellular Biology*.

[27] Moertel CG, Fleming TR, Rubin J (1982). A clinical trial of amygdalin (Laetrile) in the treatment of human cancer. *The New England Journal of Medicine*.

[28] Swain E, Poulton JE (1994). Utilization of amygdalin during seedling development of Prunus serotina. *Plant Physiology*.

[29] Bolarinwa IF, Orfila C, Morgan MR (2014). Amygdalin content of seeds, kernels and food products commercially-available in the UK. *Food Chemistry*.

[30] Barceloux DG (2009). Cyanogenic foods (cassava, fruit kernels, and cycad seeds). *Disease-a-Month*.

[31] Yang H-Y, Chang H-K, Lee J-W (2007). Amygdalin suppresses lipopolysaccharide-induced expressions of cyclooxygenase-2 and inducible nitric oxide synthase in mouse BV2 microglial cells. *Neurological Research*.

[32] Jiagang D, Li C, Wang H (2011). Amygdalin mediates relieved atherosclerosis in apolipoprotein E deficient mice through the induction of regulatory T cells. *Biochemical and Biophysical Research Communications*.

[33] Taiwo IA, Odeigah PGC, Jaja S, Mojiminiyi F (2010). Cardiovascular effects of Vernonia amygdalina in rats and the implications for treatment of hypertension in diabetes. *Researcher*.

[34] Roskoski R (2012). ERK1/2 MAP kinases: structure, function, and regulation. *Pharmacological Research*.

[35] Roskoski R (2012). MEK1/2 dual-specificity protein kinases: structure and regulation. *Biochemical and Biophysical Research Communications*.

[36] Maher P (2006). A comparison of the neurotrophic activities of the flavonoid fisetin and some of its derivatives. *Free Radical Research*.

[37] Sagara Y, Vanhnasy J, Maher P (2004). Induction of PC12 cell differentiation by flavonoids is dependent upon extracellular signal-regulated kinase activation. *Journal of Neurochemistry*.

